# Cancer‐associated *FBXW7* loss is synthetic lethal with pharmacological targeting of CDC7

**DOI:** 10.1002/1878-0261.13537

**Published:** 2023-10-22

**Authors:** Joseph S. Baxter, Rachel Brough, Dragomir B. Krastev, Feifei Song, Sandhya Sridhar, Aditi Gulati, John Alexander, Theodoros I. Roumeliotis, Zuza Kozik, Jyoti S. Choudhary, Syed Haider, Stephen J. Pettitt, Andrew N. J. Tutt, Christopher J. Lord

**Affiliations:** ^1^ The CRUK Gene Function Laboratory The Institute of Cancer Research London UK; ^2^ Breast Cancer Now Toby Robins Breast Cancer Research Centre The Institute of Cancer Research London UK; ^3^ Functional Proteomics Laboratory The Institute of Cancer Research London UK

**Keywords:** cancer, CDC7, CRISPR screen, FBXW7, synthetic lethality

## Abstract

The F‐box and WD repeat domain containing 7 (*FBXW7*) tumour suppressor gene encodes a substrate‐recognition subunit of Skp, cullin, F‐box (SCF)‐containing complexes. The tumour‐suppressive role of FBXW7 is ascribed to its ability to drive ubiquitination and degradation of oncoproteins. Despite this molecular understanding, therapeutic approaches that target defective *FBXW7* have not been identified. Using genome‐wide clustered regularly interspaced short palindromic repeats (CRISPR)‐Cas9 screens, focussed RNA‐interference screens and whole and phospho‐proteome mass spectrometry profiling in multiple *FBXW7* wild‐type and defective isogenic cell lines, we identified a number of *FBXW7* synthetic lethal targets, including proteins involved in the response to replication fork stress and proteins involved in replication origin firing, such as cell division cycle 7‐related protein kinase (CDC7) and its substrate, DNA replication complex GINS protein SLD5 (GINS4). The CDC7 synthetic lethal effect was confirmed using small‐molecule inhibitors. Mechanistically, *FBXW7*/CDC7 synthetic lethality is dependent upon the replication factor telomere‐associated protein RIF1 (RIF1), with *RIF1* silencing reversing the *FBXW7*‐selective effects of CDC7 inhibition. The delineation of *FBXW7* synthetic lethal effects we describe here could serve as the starting point for subsequent drug discovery and/or development in this area.

## Graphical abstract

Using the combination of whole genome CRISPR‐Cas9 screening, RNA interference screens, proteomic mass spectrometry profiling and small molecule inhibitors, Baxter et al. demonstrate that loss of FBXW7 in tumour cells imparts a synthetic lethal effect with CDC7 inhibition.

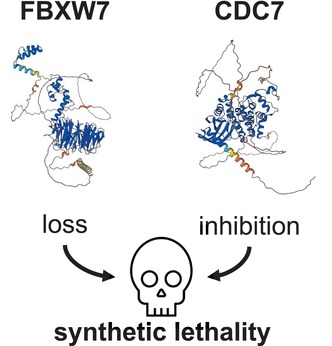

**Abbreviations**
CRISPR: Clustered regularly interspaced short palindromic repeats
CTG: Cell Titer Glo
DepMap: Dependency Map
DMSO: Dimethylsuloxide
DUB: Deubiqitinase
EdU: 5‐ethynyl‐2′‐deoxyuridine
GSEA: Gene set enrichment analysis
MOPS: 3‐(N‐morpholino)propanesulfonic acid
PAGE: Polyacrylamide gel electrophoresis
PBS: Phosphate‐buffered saline
PFA: Paraformaldehyde
PI: Propidium iodide
QN *Z*
: Quantile normalised *Z* score
RT‐qPCR: reverse transcriptase quantitative polymerase chain reaction
SDC: sodium deoxycholate
SDS: Sodium dodecyl sulphate
SEM: Standard error of the mean
sgRNA: short guide RNA
shRNA: short hairpin RNA
siRNA: short interfering RNA
SpCas9: *Streptococcus pyogenes* Cas9 enzyme
TCEP: tris‐2‐carboxyethyl phosphine
TEAB: triethylammonium bicarbonate
TMT: Tandem mass tag

## 1 Introduction

The *FBXW7* tumour suppressor gene (aka *Fbw7, Sel10, hCDC4, hAgo*, human Chr. 4q32) encodes three FBXW7 isoforms (α, β, and γ) via the use of different promoters; each isoform has a unique N‐terminal region but a common C‐terminal region [1]. FBXW7 proteins form the substrate recognition subunit of the cullin‐RING family of SCF complexes that mediate E3 ubiquitin ligation [1]. SCF complexes include the RING domain protein RBX1 (aka ROC1), the adaptor protein SKP1, a scaffold CUL1 and a F box substrate recognition protein [2]. These latter proteins include an F‐box domain in the N‐terminal region that binds SKP1‐CUL1 and protein‐interaction domains in C terminus that drive substrate binding. F‐box proteins are classified into three subclasses based on their differential C‐terminal sequence: (a) FBXW proteins that contain a WD40‐repeat domain in the C terminus; (b) FBXL proteins which contain a leucine‐rich–repeat domain; and FBXO proteins which have either no definable domain or a domain other than W‐ or L‐type C termini [3]. *FBXW7* is the most recurrently mutated F‐box gene in human cancer, acting as a tumour suppressor, being commonly mutated, deleted or hypermethylated across cancer types, notably endometrial (~ 20%) and colorectal (17.5%) subtypes [4, 5, 6, 7, 8, 9, 10]. In part at least, the tumour suppressive role of FBXW7 has been ascribed to its ability to drive the ubiquitination and degradation of oncoproteins including c‐MYC [11, 12, 13], NOTCH [14, 15, 16], c‐JUN [17, 18], Cyclin E [7, 10, 19] and MCL1 [1], as well as pro‐aneuploidy factors such as Aurora A [20, 21]. Some of these ubiquitination events are opposed by the activity of the deubiquitinase (DUB) USP28; for example, c‐MYC stability in human tumour cells is USP28 dependent and USP28 binds to MYC through a nuclear interaction with FBW7α [22].

Low FBXW7 expression is often associated with poor prognosis and resistance to many chemotherapeutics including platinum salts [23, 24], 5′fluorouracil [25], taxanes [26, 27] and doxorubicin [28]. Despite some understanding of how FBXW7 defects cause aberrant SCF complex function, only a few studies have identified targets that selectively target FBXW7‐defective tumour cells. For example, a number of approaches have been suggested that elicit FBXW7 synthetic lethality, including targeting of the integrated stress response [29], exposure to the multi‐kinase inhibitor sorafenib [30] or sensitivity to inhibition of the kinase GAK [31]. To date, a genome‐wide assessment of candidate targets in FBXW7 defective tumour cells has not been made. To address this, we describe here, efforts to identify FBXW7 synthetic lethal effects using a combination of genome‐wide clustered regularly interspaced short palindromic repeats (CRISPR)‐Cas9 and RNA interference genetic perturbation screens, used in tandem with mass spectrometry‐based proteomic profiling.

## 2 Materials and methods

### 2.1 Cell lines

MCF10A *TP53* ^*mutant*^ cells were grown in Dulbecco's Modified Eagle Medium/Nutrient Mixture F‐12 (DMEM/F12, Gibco, Billings, MT, USA) supplemented with 5% horse serum; EGF (20 ng·mL^−1^); hydrocortisone (0.5 mg·mL^−1^); cholera toxin (100 ng·mL^−1^); and insulin (10 μg·mL^−1^) and purchased from Horizon (Cat# HD + 101‐005, RRID: CVCL JM25). DLD1 FBXW7^−/−^ (Cat# HD R00‐005, RRID: CVCL_HD61) and HCT116 FBXW7^−/−^ (Cat# HD R02‐013, RRID: CVCL_HD78) and FBXW7 wild‐type DLD1 (RRID: CVCL_0248) and HCT116 (RRID: CVCL_0291) cells were also obtained from Horizon and cultured according to the supplier's instructions. Tissue culture was carried out under typical conditions (37 °C, 5% CO_2_). Mycoplasma testing was carried out every 4 passages, as was cell line identity, assayed using the GenePrint10 system (Promega, Madison, WI, USA). We can confirm that each of the cell lines used was authenticated in the past 3 years.

### 2.2 CRISPR cell line derivation and characterisation

Edit‐R gene editing methods (Horizon, Waterbeach, UK) were used to engineer MCF10A *FBXW7* mutant cells. In brief, cells were seeded at a density of 1 × 10^6^ cells per well in a 6‐well plate, along with 40 μm Edit‐R Cas9 nuclease protein NLS (Horizon), 20 μm crRNA, 10 μm tracrRNA and Lipofectamine™ CRISPRMAX transfection reagent (Cat #CMAX00003, Thermo Fisher Scientific, Waltham, MA, USA). The transfection was performed as per the manufacturer's instructions. A set of 3 crRNA (SQ‐004264‐01‐0002, Horizon) to target *FBXW7* was utilised. 96 h following transfection, cells were seeded in antibiotic‐free media at a density of one cell per well into 96‐well plates, using a FACS sorter (BD Symphony S6, Franklin Lakes, NJ, USA). To assess for successful gene targeting events, genomic DNA surrounding the guide target site was amplified using forward 5′‐AGGGCCCAAATTCACCAATA‐3′ and reverse 5′‐TAACTGGAGGCGAGGAGAAC‐3′ primers. PCR products were subsequently cloned into pCR‐TOPO‐blunt (Cat #450245, Thermo Fisher Scientific) afterwhich Sanger sequencing of the inserts was performed.

### 2.3 Reagents

AZD‐6783, CCT245737, XL413, TAK‐931, Etoposide were purchased from Selleckchem (Houston, TX, USA). Antibodies used for western blotting include: anti‐Myc (ab32072, 1 : 2000), anti‐CCNE1 (CST #4129, 1 : 1000), anti‐ABCB1 (CST #12683, 1 : 1000), anti‐FBXW7 (Bethyl #A301‐720A, 1 : 1000), anti‐Lamin A/C (CST #4777, 1 : 2000), anti‐Histon 3 (CST #4499, 1 : 1000), anti‐RIF1 (CST #95558, 1 : 1000), anti‐actin (CST #4970, 1 : 2000).

### 2.4 CRISPR screening

Genome‐wide CRISPR screens were conducted as described in [32]. Doxycycline inducible SpCas9‐expressing (Horizon, #CAS11229) cells were generated by transduction (Dharmacon, Waterbeach, UK), followed by Blasticidin selection (7 μg·mL^−1^). SpCas9 expression was confirmed via western blot. To avoid multiple sgRNA integrations per cell, inducible SpCas9 expressing cells were transduced with a previously published and validated genome‐wide human lentiviral library (Kosuke Yusa Human GW CRISPR guide RNA library V1 [33]) at an MOI of 0.3 and a representation of 1000 cells per sgRNA was maintained throughout the screen. After puromycin selection (1 μg·mL^−1^), cells were incubated with doxycycline for 3 days to induce SgCas9 and harvested at day 0 (T0) and on day 14 (T1). The DNeasy Blood and Tissue Kit (Qiagen, Venlo, Netherlands) was used to extract genomic DNA from the T = 0 and T = 1 samples as per the manufacturer's instructions. sgRNA sequence were amplified with Q5 polymerase (NEB) with the following primers Forward 5′‐ACACTCTTTCCCTACACGACGCTCTTCCGATCTCTTGTGGAAAGGACGAAACA and Reverse 5′‐TCGGCATTCCTGCTGAACCGCTCTTCCGATCTCTAAAGCGCATGCTCCAGAC [34]. The PCR products sequenced on HiSeq2500 and the data was analysed as previously described. Raw CRISPR screen data can be found on ArrayExpress under the E‐MTAB‐12939 accession number. Pathway annotation of the CRISPR‐Cas9 screen data was carried out using Enrichr as described in [35].

### 2.5 *In vitro* cell survival assays

Cells were seeded into 384‐well plates at a density of 300 cells per well in 50 μL of complete growth media. Following 24 h, different amounts of inhibitor (or vehicle) were dispensed into the media using an Echo 500 liquid handler (Beckman, Franklin Lakes, NJ, USA). Cells were incubated with drug for a total of 5 days. Cell Titer Glo (CTG) Luminescent Cell Viability Assay (Cat# G7572, Promega) was used to estimate the cell viability. This was carried out by removing the media from the wells, followed by the addition of 20 μL CTG (diluted 1 : 4 with 1X PBS). Plates were then incubated for 10 min at room temperature on a rocking platform, protected from light. Finally, luminescence was measured using a microplate reader (Victor X5, Perkin Elmer, Waltham, MA, USA).

### 2.6 Cellular proliferation and apoptosis assays

To assess proliferation, 10 000 cells were seeded into wells of a 24‐well. Growth was monitored every 8 h on the Incucyte SX5 using a 4× objective. Each data point represents the mean and SD of 8 replicate images. To assess drug sensitivity, 1000 cells were seeded into the wells of a 96‐well plate. Following 24 h DMSO or 5 μm XL‐413 was added to the media. Growth was monitored using the Incucyte SX5 using a 10× objective for 1 week. The mean percent confluence (estimated from 4 images) and standard deviation was plotted using graphpad prism (New York, NY, USA). To assess rates of apoptosis, 1000 cells were seeded into the wells of a 96‐well plate. Following 24 h DMSO or 5 μm or 10 μm XL‐413 was added to the media containing Incucyte Annexin V Red stain. 48 h later the proportion of apoptotic cells (apoptotic count/confluence) was estimated using the Incucyte SX5 with a 10× objective and imaging every 3 h.

### 2.7 Western blotting

For FBXW7, MYC, ABCB1, CCNE1 and actin cellular pellets were lysed (20 mm HEPES pH 7.9, 0.4 m NaCl, 1 mm EDTA and protease inhibitors), sonicated and incubated at 4 °C for 30 min with rotation; subsequently the lysates were cleared with 10 min centrifugation at 13 000 ***g*** and denatured with 4x NuPAGE loading dye. For RIF1 and other nuclear proteins, the cellular pellets were pre‐extracted by washing twice with 0.1% TritinX‐100 containing phosphate‐buffered saline (PBS) and subsequent lysed in RIPA Buffer (Abcam, Cambridge, UK) supplemented with 400 mm NaCl, 250 U benzonase (Sigma, St. Louis, MO, USA) and cOmplete™ Mini Protease Inhibitor Cocktail (Roche, Basel, Switzerland). Following sonication, samples were incubated at 4 °C for 30 min with rotation; subsequently, the lysates were cleared with 10 min centrifugation at 13 000 ***g*** and denatured with 4x NuPAGE loading dye. 50 μg of total protein was separated on a NuPage Novex 4–12% gradient precast gel (Thermo Fisher Scientific, NP0321BOX) using MOPS SDS running buffer (Thermo Fisher Scientific, NP0001). Separated proteins were transferred onto a nitrocellulose membrane (GE Healthcare) at 100 volts for 1 h at room temperature. 5% milk, made up in tris‐buffered saline (TBS)‐0.1% Tween buffer (TBS‐T), was used to block the membrane and incubate the primary (overnight at 4 °C) and secondary (1 h at room temperature) antibodies. Protein bands were visualised using the Licor Odyssey Fc imaging system.

### 2.8 qRT‐PCR

The Qiagen RNeasy kit was used to extract total RNA from cells and 1 μg of RNA was reverse transcribed with either the High‐capacity cDNA reverse transcription kit (Cat# 4368814, Thermo Fisher Scientific) or the Promega GoScript Reverse Transcription System (#PRA5000, Promega), as per kit instructions. 25 ng cDNA was amplified with 125 nm Hs00217794_m1 FBXW7 TaqMan probe human (Cat# 4331182, Thermo Fisher Scientific) and Hs02786624_g1 GAPDH TaqMan probe human (4448489, Thermo Fisher Scientific) with TaqMan master mix. For quantification the QuantStudio 6 Flex Real‐Time PCR System (Thermo Fisher Scientific) was utilised. Fold changes in mRNA for siRNA transfected or CRISPR mutagenized cells were calculated as 2^ΔΔ*C*t^. To estimate this value the cycle threshold (Ct) for the mRNA of interest was subtracted from the Ct value of the internal control, GAPDH, to generate the ΔCt value. In this instance, both Ct values were the mean of duplicate amplifications from the same RT‐PCR reaction. The ΔCt value was then subtracted from the equivalent ΔCt value from the wild‐type or siCTRL‐treated cells to generate the ΔΔ*C*t.

### 2.9 Mass spectrometry

Proteomics analysis was performed with the SimPLIT method as previously described [36], utilizing peptide TMTpro labelling followed by high‐pH Reversed‐Phase fractionation and LC–MS analysis at the MS3 or MS2 modes. Phosphopeptide enrichment was performed in peptide fractions with the High‐Select Fe‐NTA Phosphopeptide Enrichment Kit (Thermo) using a modified 96‐well plate protocol where the binding, washing and elution steps were performed in tip columns. The flow‐throughs were used for total proteome analysis. The SequestHT and Mascot search engines were used for protein identification and quantification in Proteome Discoverer 2.4. MS1 and MS2 mass tolerances were 20 ppm and 0.5 Da (CID spectra) or 0.02 Da (HCD spectra) respectively. Static modifications were TMTpro at N‐terminus/lysine and carbamidomethyl at cysteine. Dynamic modifications were oxidation of methionine, deamidation of asparagine/glutamine and phosphorylation of serine, threonine and tyrosine. Peptides were filtered at *q*‐value<0.01 based on target‐decoy database search with the Percolator node. The IMP‐ptmRS node was used to compute phosphorylation localization probabilities.

### 2.10 siRNA gene silencing experiments

RNAi transfections were carried out in either 96‐well or 384‐well plates by reverse transfection using SMARTpool siRNA and siCON2 non‐silencing controls (Horizon). Cells were transfected with 20 nM siRNA with 5% RNAiMAX (Thermo Fisher Scientific) in 50 μL (384‐well plate) or 500 μL (6‐well plates). Following 2–3 days post transfection cells were collected for extracting lysates or viability assays. siRNA library, individual siRNAs (M‐027983‐01) and SMART pool (MQ‐027983‐01) for RIF1 were ordered from Dharmacon.

### 2.11 Cell cycle analysis

Cells were seeded into a 6‐well plate at 50% density (~ 150 000 cells/well). After 24 h media was replaced with media containing drug or DMSO and left to incubate for a further 24 h. During the last 2 h of drug treatment 20 μm 5‐ethynyl‐2′‐deoxyuridine (EdU, ThermoFisher) was added to the culture medium. Cells were harvested by trypsinisation, washing in ice cold PBS before fixation in 70% ethanol at 4°C overnight. The Click‐iT™ Plus EdU Alexa Fluor™ 647 Flow Cytometry Assay Kit (Invitrogen, Waltham, MA, USA) was used to stain the cells and was carried out as per the manufacturer's instructions. In brief, cells were permeabilised in 1 x saponin diluted in PBS before being stained with Alexa Fluor™ 647. Cells were stained with PI for DNA content with FxCycle™ PI/RNase Staining Solution (ThermoFisher). Detection of EdU staining and PI was performed on a BD LSR II flow cytometer (BD biosciences) and FlowJo (BD) FACS analysis software was used to examine cell cycle profiles.

### 2.12 EdU labelling

Cells were seeded onto pre‐coated coverslips (P2636, Sigma Aldrich) in a 24‐well plate at a density of 75 000 cell per well. After 24 h, EdU was added to the media at a concentration of 25 μm and incubated for 10 min. Following 3x washes with PBS, cells were fixed in 4% (v/v) PFA/PBS for 10 min at room temperature. Following 3x washes with PBS, permeabilization was carried out with 0.2% (v/v) Triton X‐100 for 20 min at RT. Afterwards, the click reaction was carried out by adding the click reaction buffer (100 mm Tris pH 8, 4 mm CuSO_4_, 100 mm sodium ascorbate, 50 μm biotin‐azide) to the samples and incubating at 37 °C for 2 h. Following 3x washes with PBS and counterstaining with DAPI (Life Technologies, Carlsbad, CA, USA, 1 : 50 000 dilution) the cells were mounted using fluorescence mounting medium (S3023, Dako, Santa Clara, CA, USA). Fluorescent images were acquired using a Delta Vision widefield microscope (GE Healthcare Life Sciences) and multiple different fields were imaged per sample (60× objective).

## 3 Results

### 3.1 Generation of a novel FBXW7 isogenic cell line model

**Fig. 1:**
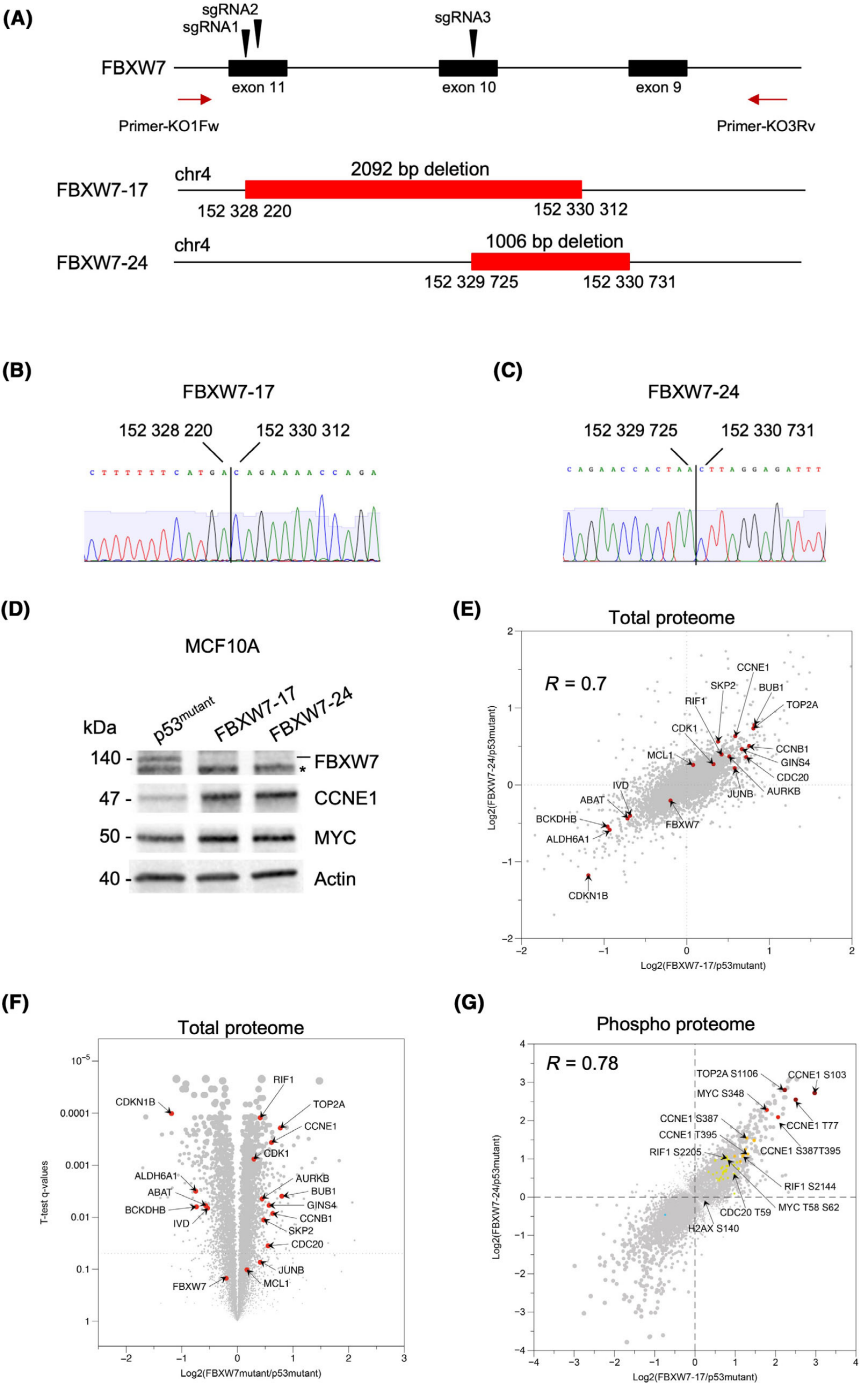
Generation and characterization of novel FBXW7 isogenic cell line models. (A) Schematic describing binding site of FBXW7‐targeting short guide (sg)RNA (black arrows) used to generate FBXW7 defective subclones in MCF10A p53^mutant^ cells. MCF10A p53^mutant^ cells with doxycycline‐inducible SpCas9 were transfected with FBXW7‐tageting sgRNAs. After doxycycline exposure to induce Cas9, daughter clones with deletions in *FBXW7* were identified using a diagnostic PCR with Primer‐KO1FW and Primer‐KO3Rv (red arrows). The amplified PCR products for clone 17 and 24 showed deletions of various sizes (indicated with red box) in the FBXW7 gene (Fig. S1A). (B, C) Sanger sequencing validation of the deletion breakpoints of clones 17 (B) and 24 (C) using PCR primers described in (A). Both clones showed clear transition between the two positions annotated with their genomic coordinates. (D) Western blot analysis of FBXW7, CCNE1 and MYC in MCF10A TP53^−/−^ FBXW7^mutant^ cells. *n* = 1 experiment. Uncropped blot scans are presented in Fig. S1C. *non‐specific band. (E) Total proteome comparison of the FBXW7 mutant cones. The scatter plot shows the Log2(FBXW7^mutant^/p53^mutant^) for FBXW7‐17 on the *x*‐axis and FBXW7‐24 on the *y*‐axis, respectively. The presented values are an average of *n* = 2 experiments. A number of significantly up‐ and down‐regulated proteins are annotated, including known FBXW7 substrates. Detailed lists of these proteins are provided in Table S1. The correlation coefficient between the abundances in the total proteomes between both clones was *R* = 0.7, indicating that the two proteomes are strongly correlated. (F) A volcano plot of whole proteome mass spectrometry measurements with averaged Log2(FBXW7^mutant^/p53^mutant^) on the *x*‐axis and *t*‐test *q*‐values on the *y*‐axis. The presented values are an average of *n* = 2 experiments. (G) Phospho‐proteome of the FBXW7^mutant^ cells displayed as in (E). The presented values are an average of *n* = 2 experiments. The arrows annotate a number of know FBXW7 targets phospho‐peptides (e.g. CCNE1 and MYC); in addition RIF1 phospho‐peptides are shown. The correlation coefficient between the abundances in the phospho‐proteomes between both clones was *R* = 0.78, indicating that the two phospho‐proteomes are strongly correlated.

To identify FBXW7 synthetic lethal effects, we first generated isogenic FBXW7 wild‐type and defective cell lines from a non‐tumour epithelial cell line with a previously engineered p53 mutation, MCF10A p53^mutant^. We then used these in genome‐wide CRISPR‐Cas9 synthetic lethal screens. We used a p53 mutant derivative of MCF10A for these experiments, for two reasons: (a) to better model tumour cells with p53 pathway dysfunction; and (b) so that in later CRISPR‐Cas9 genetic screens, mutations that often impair cellular fitness by invoking p53‐mediated cell cycle checkpoints are better tolerated. We also reasoned that imposing a FBXW7 defect on a non‐tumour epithelial cell line, as opposed to a tumour cell line with likely pre‐existing alterations in a number of FBXW7 substrates, would maximise the possibility of identifying FBXW7 synthetic lethal effects. To generate FBXW7 mutant daughter clones from MCF10A p53^mutant^ cells, we carried out CRISPR‐Cas9 mutagenesis of *FBXW7* using short guide (sg)RNAs designed to target exons 10 and 11 of *FBXW7* (Fig. 1A) and generated clones from single cells for PCR screening. This approach generated two FBXW7 mutant subclones, FBXW7‐17 and FBXW7‐24, each of which had deletions within the FBXW7 gene (Fig. 1A, Fig. S1A). Sanger sequencing indicated that FBXW7‐17 contained a 2093 bp deletion within *FBXW7* (between genomic coordinates chr4:152328220 and 152330312), whilst FBXW7‐17 contained a 1007 bp deletion (between genomic coordinates chr4:152329725‐152330731); both of these deletions removed coding exons of the gene (Fig. 1A–C). RT‐qPCR showed that FBXW7 mRNA levels were significantly reduced in both FBXW7‐17 and FBXW7‐24 cells (Fig. S1B) and western blotting confirmed a reduction in FBXW7 protein (Fig. 1D and Fig. S1C). In addition, the levels of cMyc and Cyclin E1 (CCNE1), known FBXW7 substrates, were upregulated in FBXW7‐17 and FBXW7‐24 cells (Fig. 1D and Fig. S1C). We also used mass spectrometry to define the total and phospho‐proteome of FBXW7‐17 and FBXW7‐24 cells and compared these to FBXW7 wild‐type MCF10A p53^mutant^ cells (Tables S1 and S2). The changes in the abundance of proteins and phosphorylation events in FBXW7‐17 and FBXW7‐24 cells were similar (Fig. 1E–G; correlation coefficient *R* > 0.7). When compared to wild‐type cells, both FBXW7‐17 and FBXW7‐24 cells had increased levels of known FBXW7 substrates and phosphodegrons, such as those in CCNE1 and MYC (Fig. 1E–G and Fig. S1D) as well as increases in predicted FBXW7 phosphodegrons [37], such as those in CCNE1 and RIF1 (Fig. S1D,E). Gene Set Enrichment Analysis (GSEA) of the upregulated proteins (Table S3) and phosphorylation events (Table S4) in FBXW7 defective cells showed that proteins involved in cell cycle control (adjusted *P*‐value = 0.002) and synthesis of DNA (*P*‐value = 2.10^−4^) were amongst the most enriched processes. Surprisingly, amongst the down‐regulated processes in the FBXW7^mutant^ cells, there was a noticeable enrichment in proteins regulating the metabolism of branched and other amino acids (Fig. 1E and Table S5).

### 3.2 A genome‐wide CRISPR‐Cas9 mutagenesis screen identifies FBXW7 synthetic lethal effects

**Fig. 2:**
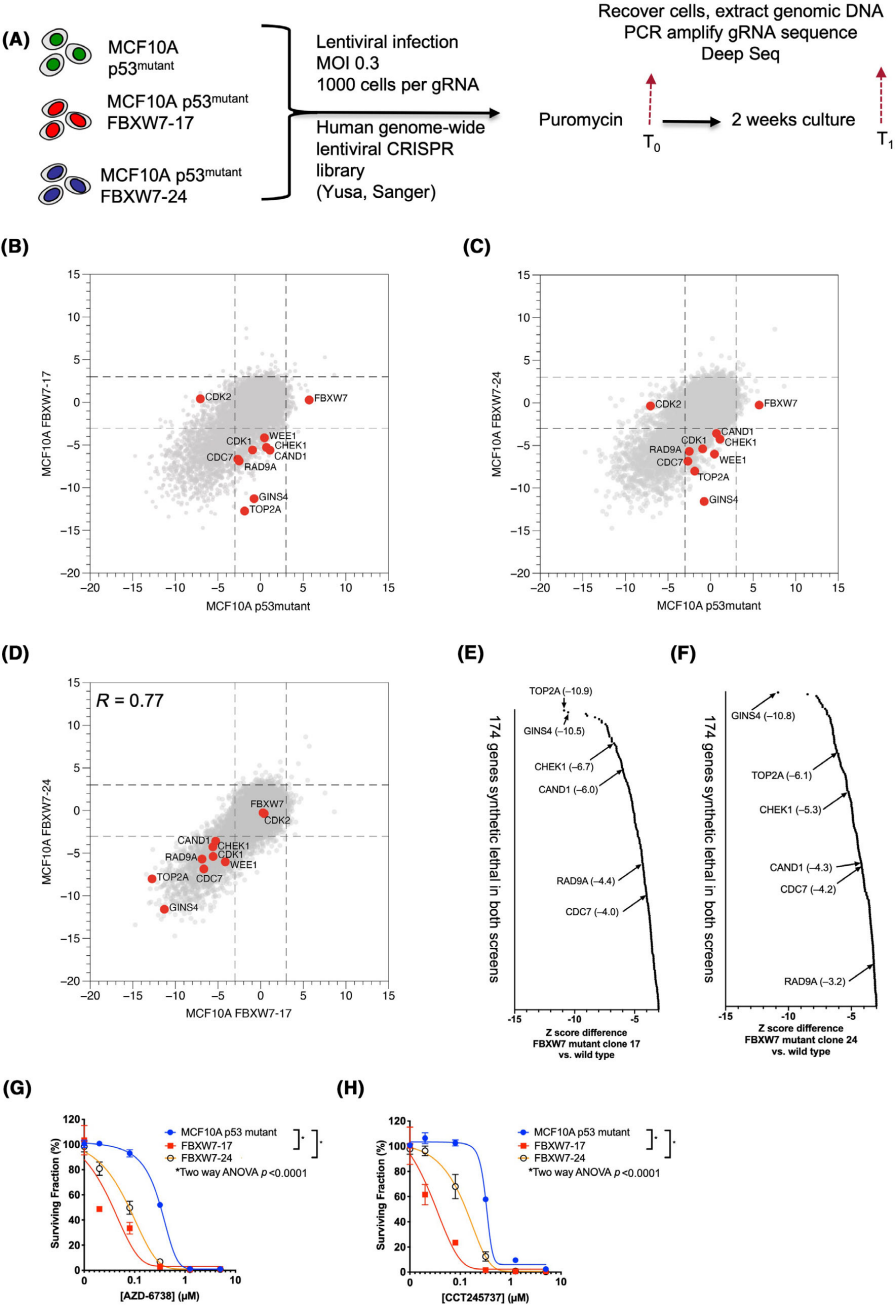
Identification of FBXW7 synthetic lethal effects from a genome‐wide CRISPR‐Cas9 mutagenesis screen. (A) Schematic describing the genome‐wide CRISPR‐Cas9 screen workflow. (B) Scatter plot illustrating gene‐level *Z* scores from CRISPR‐Cas9 screen in MCF10A FBXW7‐17 vs. p53 mutant cells. Each screen arm was sequenced as a single sample. Highlighted are several of the FBXW7 synthetic lethal genes in this clone. (C) Scatter plot illustrating gene‐level *Z* scores from CRISPR‐Cas9 screen in MCF10A FBXW7‐24 vs. p53 mutant cells. Each screen arm was sequenced as a single sample. Highlighted are several of the FBXW7 synthetic lethal genes in this clone. (D) Comparison of synthetic lethal effects in clone 17 vs clone 24. *Z* score differences from clone 17 vs clone 24 screen are shown. Each screen arm was sequenced as a single sample. The correlation coefficient between the *Z*‐scores in both clones was *R* = 0.77, showing strong correlation. (E, F) A waterfall plot, showing the most profound FBXW7 synthetic lethal effects identified in clone 17 (E) and clone 24 (F) screens. Arrows indicate the genes described in the main text. (G, H) Dose response survival curves using either the ATR inhibitor AZD6738 (G) or the CHK1 inhibitor CCT245737 (H). Cells were constantly exposed to media containing inhibitor for 5 days after which cell viability was estimated by Cell Titer Glo. Error bars represent SEM from *n* = 3 experiments; *Two‐way ANOVA *P*‐value < 0.0001.

To identify FBXW7 synthetic lethal effects, we generated Cas9‐expressing versions of MCF10A p53^mutant^, FBXW7‐17 and FBXW7‐24 cells and then mutagenised these with a genome‐wide short guide (sg)RNA library designed to target 18 006 protein coding genes (90 709 sgRNAs, Fig. 2A). In totality, 300 × 10^6^ cells were transduced at a multiplicity of infection of 0.3 (to ensure < 1 sgRNA per cell), resulting in each sgRNA infecting at least 1000 cells, a representation that was maintained throughout the experiment. After removing non‐transduced cells and removing a fraction of the cell population for later analysis (T_0_ sample), the resultant cell population was cultured for 2 weeks at which point DNA from surviving cells was recovered (T_1_ sample). Using deep sequencing, we estimated the relative enrichment or depletion of sgRNAs from T_0_ vs. T_1_ samples and used this data to calculate gene level quantile normalised *Z* scores (QN *Z*) for each gene in each cell line; in this case, genes with negative *Z* scores impaired the fitness of cells, with a *Z* score threshold of < −3 being used to identify profound effects. Relevant to the performance of the CRISPR screen, all cell lines had a similar growth rate (Fig. S2A) and were equally sensitive to puromycin selection as they did not overexpress the drug efflux pump ABCB1 (Fig. S2B and Fig. 1C). The full set of QN *Z* scores for each cell line is provided in Table S6. When examining the screen data, we noted that CRISPR‐Cas9 targeting of *FBXW7* caused a short‐term fitness advantage in FBXW7 wild‐type MCF10A p53^mutant^ cells (*Z* score = 5.7) but had negligible effects in FBXW7‐17 and FBXW7‐24 cells (*Z* scores of 0.28 and − 0.26 respectively, Fig. 2B,C), consistent with FBXW7‐17 and FBXW7‐24 cells being FBXW7 defective. When we compared synthetic lethal effects in FBXW7‐17 vs. FBXW7‐24 screens, we found these to be highly reproducible (Fig. 2D, correlation coefficient *R* = 0.77). To identify the most reproducible synthetic lethal effects, we selected those genes that fulfilled the following criteria in both FBXW7‐17 and FBXW7‐24 screens: (a) a *Z* score difference of > 3 *Z* scores between FBXW7 wild‐type and mutant cell lines; and (b) a *Z* score of < −3 in the FBXW7 mutant cell line. This resulted in the identification of 174 candidate synthetic lethal genes (Table S7). GSEA and pathway analysis of these 174 genes identified a significant enrichment for a number of partially overlapping gene sets including those associated with the G_1_/S transition (*P* = 6.10^−7^), origin regulation to post‐replicative state (*P* = 10^−6^), cell cycle and mitosis pathways (*P* = 10^−16^), transcription and mRNA processing (*P* = 5.10^−12^), translation (*P* = 5.10^−15^) and DNA damage control and checkpoints (*P* = 4.10^−6^, Table S8).

Amongst the most profound synthetic lethal effects (Fig. 2E,F), we noted *TOP2A*, which encodes DNA topoisomerase 2 α (TopoIIα) and controls DNA topology by transient breakage and subsequent re‐joining of DNA strands. Interestingly, FBXW7 has previously been implicated as the E3 ligase responsible for targeted TopoIIα degradation, with loss of FBXW7 driving an increase in TopoIIα levels [38, 39]. Consistent with this, in our proteomic data we observed that TOP2A (and phospho‐S1106) were amongst the most increased proteins (Fig. 1E–G). We also noted the FBXW7 interacting protein CAND1 (Cullin‐associated NEDD8‐dissociated protein 1 [40]) in our list of FBXW7 synthetic lethal interactions. CAND1 acts a protein exchange factor that recruits new F box proteins to SCF complexes [40]. One plausible mechanism to explain the FBXW7/CAND1 synthetic lethality might be that in the absence of FBXW7, CAND1 is normally required to supplement SCF complexes with alternative F box proteins [40]. Synthetic lethal interactions with proteins involved in ATR kinase signalling were also seen in our analysis, notably RAD9A and CHEK1. In subsequent validation experiments, we noted that both FBXW7‐17 and FBXW7‐24 cells were more sensitive to the clinical ATR inhibitor AZD‐6738 (Ceralasertib, AstraZeneca, Cambridge, UK) as well as to a small molecule CHEK1 inhibitor, CCT245737 (Fig. 2G,H). These results were consistent with prior observations [41]. Amongst other functions associated with genome stability, ATR also plays a key role in controlling the licencing of replication origins once DNA damage has occurred [42]. We also noted a number of synthetic lethal interactions associated with proteins involved in the licencing of replication forks, including CDC7 kinase (which itself has been implicated as a FBXW7 target [43]); the CDC7 substrate GINS4 [42] was also a strong synthetic lethal hit and was also increased in the proteome of FBXW7^mutant^ cells (Figs 1E and 2E,F). CDC7's role in replication largely focuses on the activity of the Cdc45, MCM and GINS (CMG) proteins at pre‐replication complex [44]; specifically CDC7 controls CMG helicase activity [44, 45] by phosphorylating MCM4 [46]. Opposing the function of CDC7, is RIF1, which acts with PP1 to dephosphorylate CDC7 kinase sites [47]. ATR is also known to phosphorylate MCM complexes as a means to control fork firing [48].

### 3.3 FBXW7/CDC7 synthetic lethality is a relatively penetrant effect

**Fig. 3:**
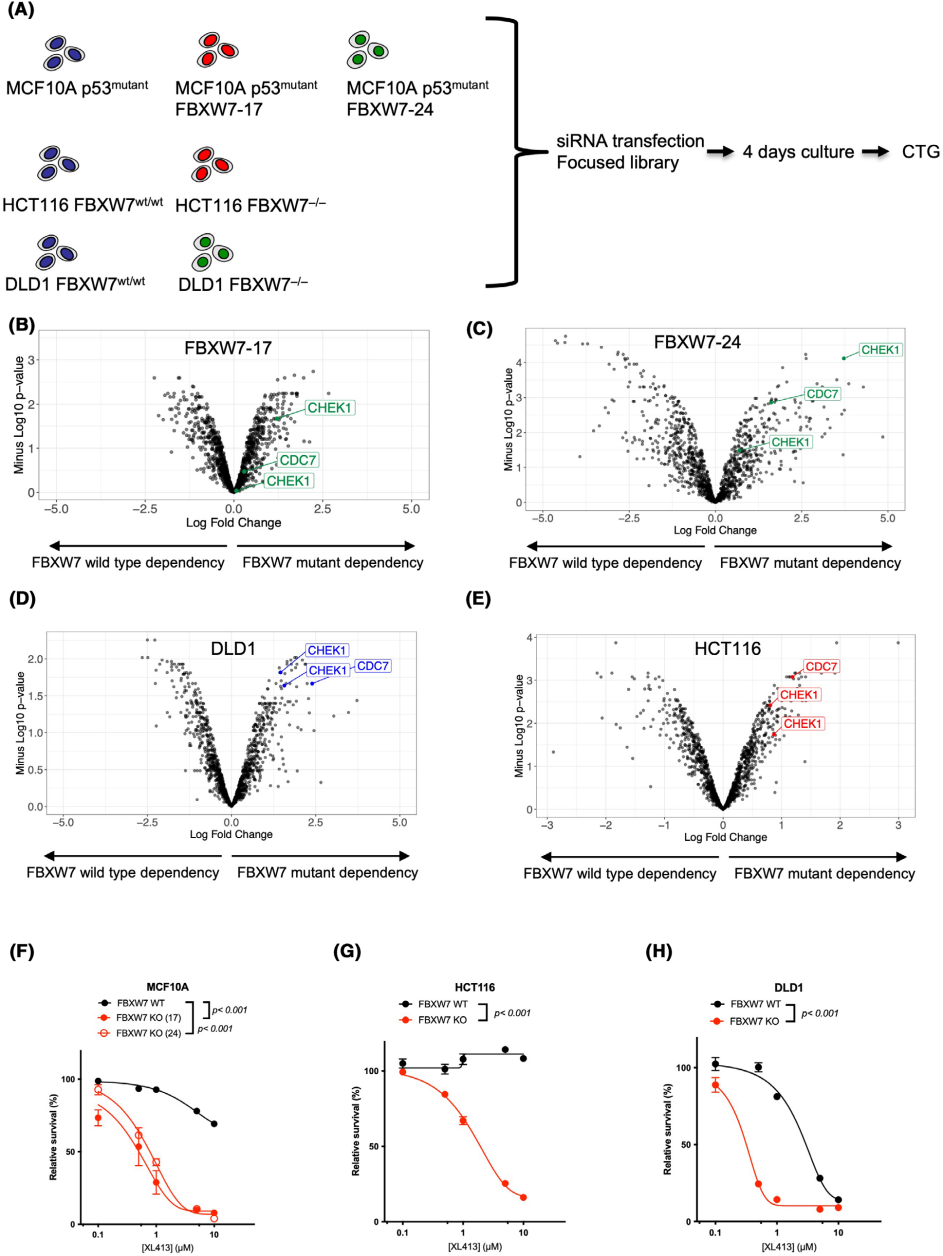
FBXW7 vs. CDC7 synthetic lethality is reproduced in isogenic cell lines. (A) Schematic describing validation screen workflow using three different isogenic systems, MCF10A p53^mutant^ isogenic cells, HCT116 isogenic cells and DLD1 isogenic cells. (B–E) Scatter plot illustrating synthetic lethal effects from validation screens in MCF10A FBXW7‐17 (B), MCF10A FBXW7‐24 (C), DLD1 (D) and HCT116 (E) cells. Each point represents an average of *n* = 3 experimental replicates. CHEK1 siRNA is present twice in the siRNA library (gene list provided in Table S9). (F–H) Dose response survival curves using either the CDC7 inhibitor XL413 in MCF10A FBXW7‐17 and ‐24 cells (F), in FBXW7 isogenic HCT116 cells (G) and in FBXW7 isogenic DLD1 cells (H). Cells were constantly exposed to media containing inhibitor for 5 days after which cell viability was estimated by Cell Titer Glo. Error bars represent SEM from *n* = 3 experiments; *P*‐values were determined by two‐way ANOVA.

Whilst a number of cancer‐associated synthetic lethal interactions have been identified, many of these are private to a very specific molecular context, have incomplete penetrance and fail to be reproduced in other, model systems that have the same cancer driver gene, but are otherwise molecularly distinct [49]. For example, when we assessed the relationship between CDC7 gene dependency and FBXW7 mRNA expression in 43 colorectal tumour cell lines profiled using genome‐wide shRNA libraries as part of the DepMap initiative, we noted a positive correlation, Pearson's correlation *R* = 0.3 and a linear regression *P* = 0.051 (i.e. tumour cell lines with reduced FBXW7 mRNA exhibited greater dependency upon CDC7, Fig. S2C). To extend these observations, we carried out secondary genetic screens in three FBXW7 isogenic systems: (a) the MCF10A p53^mutant^ isogenics described above; (b) previously described HCT116 colorectal tumour cells with/without gene targeting of *FBXW7* (HCT116 FBXW7^wt/wt^ and HCT116 FBXW7^−/−^ [8]); (c) previously described DLD1 colorectal tumour cells with/without gene targeting of *FBXW7* (DLD1 FBXW7^wt/wt^ and DLD1 FBXW7^−/−^ [8]). Given the observation that CHEK1 and CDC7 kinases appeared synthetic lethal in our genome‐wide screen, in these subsequent screens we used 384‐well plate arrayed short interfering (si)RNA to silence a total of 1213 genes, including the kinome. Cells were reverse transfected with the siRNA library and then cultured for a subsequent 4 days after which cell viability was estimated using Cell Titre Glo reagent (Fig. 3A). The analysis of these arrayed screens is included in Table S9. In all three isogenic systems, the synthetic lethal effects associated with CHEK1 and CDC7 were seen, indicating that these were relatively penetrant effects (Fig. 3B–E). The fact that these were also elicited using siRNA, as opposed to CRISPR‐Cas9 targeting used in the genome‐wide screen, suggested that these particular synthetic lethal effects were also unlikely to be an artefact or private to an individual form of gene perturbation. Furthermore, when we used a previously validated CDC7 small molecule catalytic inhibitor XL413 [50] we replicated the FBXW7 synthetic lethal effect in all three isogenic systems (MCF10A p53^mutant^, DLD1 and HCT116, Fig. 3F–H). We confirmed this observation using a second CDC7 inhibitor, TAK‐931 (Fig. S3A–C). CDC7 inhibition impaired the proliferative capacity of FBXW7^mutant^ cells (Fig. S3D–F) and also caused apoptosis (Fig. S3G–I). We note that HCT116 cells are p53 wild type, whereas DLD1 cells are p53 mutant (p.S241F), as are the MCF10A cells used to originally identify the CDC7 synthetic lethality. As all three cell lines show the CDC7/FBXW7 synthetic lethality, we reasoned that it is unlikely that this synthetic lethal effect is entirely reliant upon p53 status.

Collectively, these experiments suggested that the FBXW7/CDC7 synthetic lethality was not only relatively penetrant but was also relatively independent of the mode of CDC7 inhibition.

### 3.4 CDC7 inhibitor sensitivity in FBXW7 mutant cells is RIF1 dependent

**Fig. 4:**
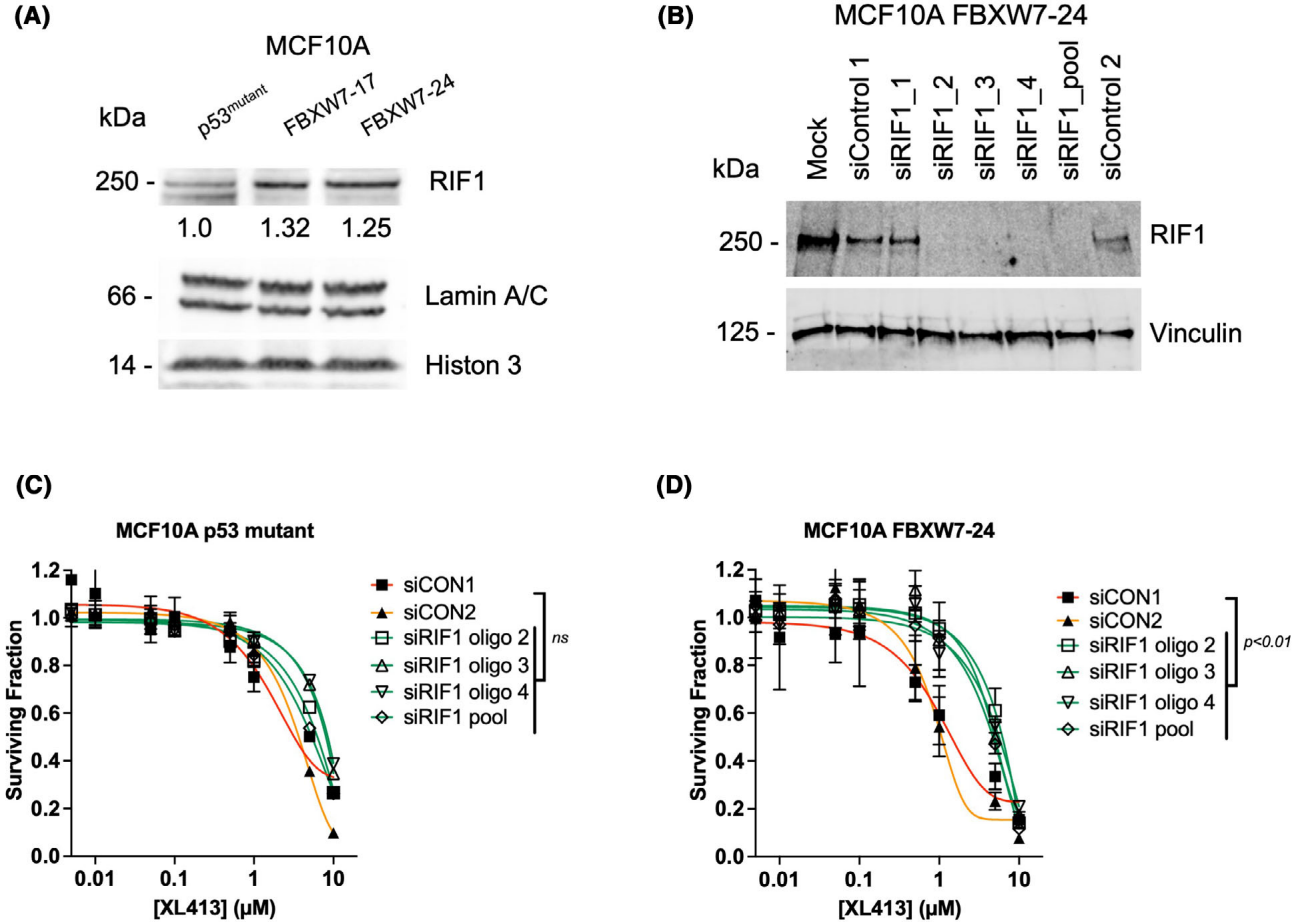
FBXW7 vs. CDC7 synthetic lethality is RIF1 dependent. (A) Western blot showing that RIF1 levels are increased in the FBXW7^mutant^ cells. Uncropped blot scans are presented in Fig. S1C. Quantification was conducted by band densitometry and normalization to the loading controls. (B) Western blot analysis of MCF10A FBXW7‐24 cells, transfected with a SMARTpool or individual siRNAs targeting RIF1, showing efficient downregulation of the target protein. (C, D). Dose response curves of MCF10A p53^mutant^ and FBXW7‐24 cells to CDC7i (XL413) upon RIF1 depletion. siRIF1_1 oligo was not used in these experiments as it does not lead to RIF1 depletion. The data points represent mean and standard deviation of *n* = 3 experiments; statistical analysis was done by two‐way ANOVA *P* < 0.001.

To determine the potential mechanism of CDC7 inhibitor sensitivity in FBXW7^mutant^ cells, we performed proteomic mass spectrometry in the MCF10A isogenic cells. Interestingly, we observed that Rap1 interacting factor, (RIF1), exhibited increased expression in both FBXW7^mutant^ cell lines, compared to parental FBXW7 wild‐type cells (Fig. 1E,F). This was interesting as RIF1 is known to oppose the function of CDC7, by acting with PP1 to dephosphorylate CDC7 kinase sites [47]. We first used western blots to confirm the upregulation of RIF1 levels in FBXW7 mutant cells (Fig. 4A and Fig. S1C). Using phosphoproteomic profiling of FBXW7 wild‐type and mutant cells, we also identified a number of phosphorylation sites within RIF1 that were upregulated (Fig. S1E), including a recently described putative FBXW7‐dependent phosphodegron [37].

**Fig. 5:**
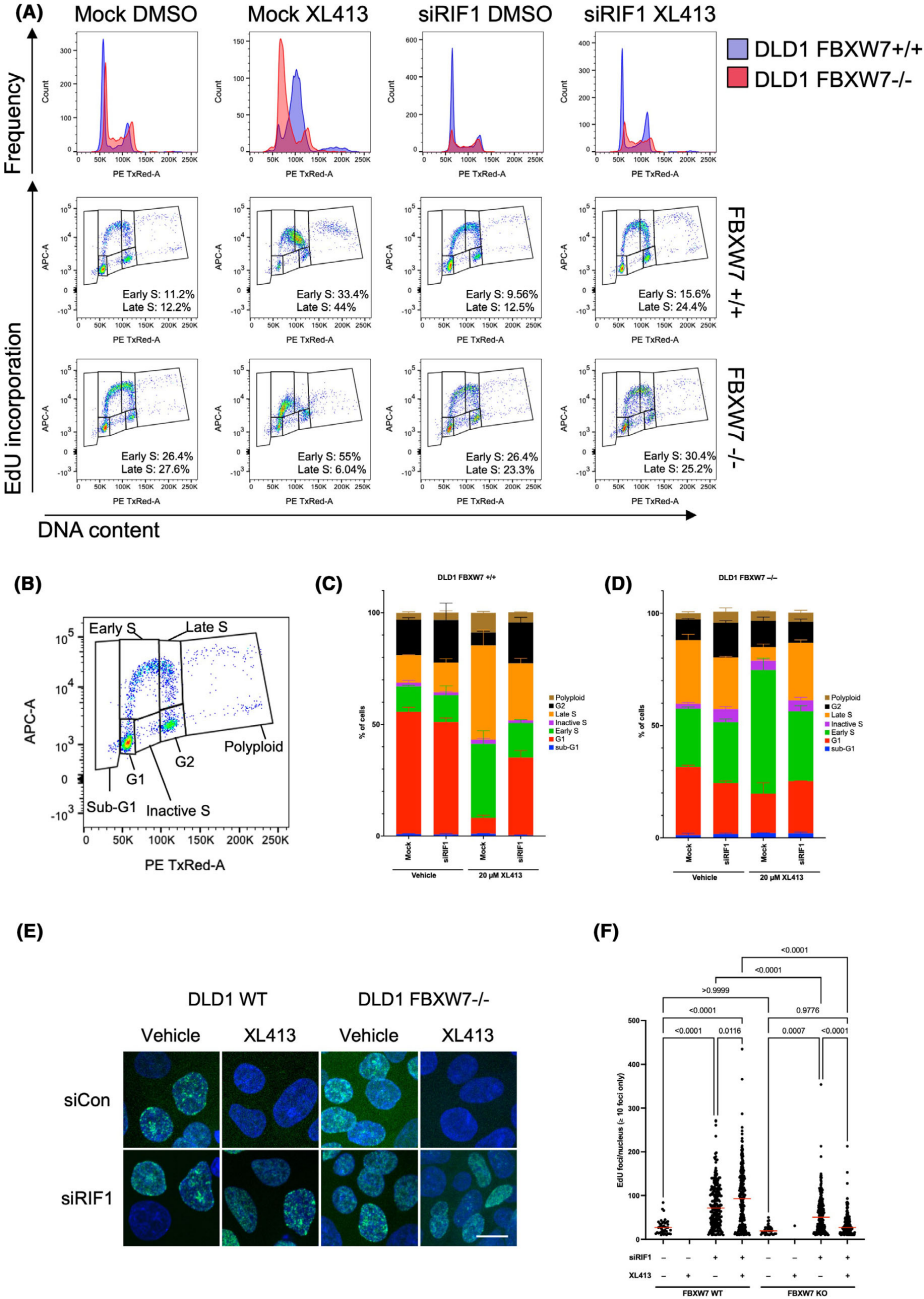
RIF1 depletion reverses the effect of CDC7i on S‐phase in FBXW7‐mutant cells. (A) FACS analysis demonstrating the effect of RIF1 depletion on the CDC7i‐induced S‐phase arrest. DLD1 FBXW7+/+ or FBXW7−/− cells were either control‐ or RIF1‐depleted for 48 h and subsequently treated with 20 μm CDC7i for 16 h; in the last 2 h of the treatment 10 μm EdU was added to the media. After fixation and click chemistry the distribution in the cell cycle was analysed by the intensity of EdU/DNA stain (see Section 2). CDC7i treatment leads to accumulation of S‐phase cells, which is reversed when RIF1 is depleted. Cell cycle profiles were overlayed for comparison of the different genotypes – DLD1 FBXW7+/+ in blue and FBXW7−/− in red, respectively. (B) A diagram of the cell cycle distribution showing the gates used to quantify early, late, and inactive S‐phase. (C, D) A quantification of the different phases of the cell cycle as presented in panel A for the parental (C) and the FBXW7−/− (D) cells. Mean and standard deviation of *n* = 3 experiments are shown. (E) Representative images from the experiment described in Fig. S5A. The scale bar represents 10 μm. (F) Quantification of the EdU foci/nucleus in cells with over 10 foci/nucleus from the experiment described in Fig. S5A. CDC7i‐only treatment results in no cells over this threshold. Analysed > 200 nuclei per conditions from *n* = 3 experiments; statistical significance was determined by ordinary one‐way ANOVA.

Consistent with RIF1 opposing the function of CDC7, [47], loss of RIF1 has previously been shown to cause CDC7 inhibitor resistance and RIF1 gene silencing reverses CDC7i‐induced accumulation of S phase cells [51]. We therefore reasoned that elevated RIF1 in FBXW7 defective cells could be responsible for CDC7 inhibitor sensitivity in FBXW7 mutant cells. We assessed a range of RIF1 siRNAs for their ability to deplete the protein (Fig. 4B) and then found that those that did mediate gene silencing partially reversed the CDC7 inhibitor sensitivity of FBXW7 mutant cells (Fig. 4C,D and Fig. S4), establishing a causal relationship between RIF1 and CDC7 inhibitor sensitivity. Consistent with these observations, our phosphoproteomic profiling indicated that phosphorylation of RIF1 S2205 is higher in FBXW7^mutant^ cells (Fig. S1E). S2205 is part of a critical interaction surface with PP1 and its phosphorylation prevents the recruitment of PP1 to replication origins, thus alleviating PP1's inhibition on replication origin firing [52]. Given these observations, we assessed the effect of RIF1 silencing on cell cycle progression of FBXW7^mutant^ cells exposed to CDC7 inhibitor. As expected, [51], CDC7 inhibitor exposure led to the accumulation of cells in S phase (Fig. 5A–D), and more specifically, an accumulation of cells in early S phase, as opposed to late S phase accumulation. Silencing of RIF1 was sufficient to reverse S phase accumulation in both FBXW7 wild‐type and mutant cells (Fig. 5C,D). We also assessed the effect of RIF1 silencing on DNA synthesis using EdU incorporation in FBXW7^mutant^ cells (Fig. S5A), finding that RIF1 silencing was sufficient to rescue the loss of EdU foci otherwise induced by CDC7 inhibition (Fig. 5E,F), suggesting RIF1 is required for CDC7i‐induced inhibition of replication. RIF1 silencing also increased the size and intensity of EdU foci (Fig. S5B,C), suggesting a global restoration of DNA synthesis.

## 4 Discussion

In summary, we describe here a genome‐wide assessment of candidate synthetic lethal effects put in place by defects in the *FBXW7* tumour suppressor. The genome‐wide CRISPR‐Cas9 screens, focussed kinome siRNA screens and total and phosphoproteomic profiles of FBXW7 isogenic cells generated in this work provide a starting point and datasets that could be used to identify additional FBXW7 synthetic lethal effects as a prelude to the eventual discovery of a clinically actionable approach to targeting cancers with defects in this tumour suppressor.

The synthetic lethal effects identified here include SCF‐related proteins such as CAND1, proteins involved in the response to replication fork stress such as ATR, RAD9A and CHEK1 but also proteins involved in replication origin firing such as CDC7. The ATR, CHEK1 and CDC7 synthetic lethal effects can be replicated using small molecule inhibitors and in addition, the CDC7 dependency of colorectal tumour cell lines with reduced FBXW7 mRNA expression in the DepMap dataset was seen. Proteomic profiling of FBXW7 isogenic cells highlighted the possibility that the FBXW7 vs. CDC7 synthetic lethal effect could be caused by upregulation of RIF1; subsequent functional experiments showed that FBXW7 vs. CDC7 synthetic lethality is indeed RIF1 dependent. In support of this notion, we also found that a critical phosphorylation event (pS2205) in RIF1 is upregulated in the FBXW7^mutant^ cells. The increase in this phosphorylation could potentially prevent PP1‐mediated inactivation of origin firing and thus alleviate a break imposed upon replication by CDC7 inhibition. Phospho‐proteomic analysis of FBXW7 isogenic cells also suggested that a FBXW7‐dependent phosphodegron in RIF1 might exist. There were several limitations of our study and areas which require further investigation. For example, when analysing shRNA data from colorectal tumour cell lines included in the DepMap dataset, we noted a relationship between CDC7 dependency and reduced expression of FBXW7; we do note that a similar relationship was not seen when tumour cell lines from other cancer histologies were taken into account (data not shown). The histology specific nature of this relationship (which is common in cancer‐related synthetic lethal effects [49]) might reflect the FBXW7/CDC7 synthetic lethal effect being more penetrant in cancer histologies such as colorectal cancer where FBXW7 acts as a tumour suppressor, than in histologies where it is not. We also note that in our analysis of DepMap data, we did not see a relationship between FBXW7 deleterious mutation and CDC7 dependency (data not shown), despite this being apparent in three different isogenic cell line pairs with mutations that ablate FBXW7 expression (MCF10A, DLD1 and HCT116). Although there might be multiple technical reasons for a failure to see such a relationship, such as the number of cell lines with deleterious FBXW7 mutations in a cell line panel and the statistical power of such an analysis, it is also possible that it is loss of FBXW7 expression (whether caused by mutation or not) that is the real driver of CDC7 synthetic lethality, and not FBXW7 mutation *per se*.

Although our data identify RIF1 as being critical for the FBXW7/CDC7 synthetic lethal effect, we have not assessed whether RIF1 is itself a direct FBXW7 substrate or whether the increased levels of RIF1 seen are the result of a more convoluted mechanism that is put in place by FBXW7 dysfunction. Site‐directed mutagenesis of the putative RIF1 phosphodegron might resolve this question. It is also possible that the increased expression of RIF1 could reflect a homeostatic response to replication fork stress imparted upon cells by dysregulation of other FBXW7 substrates such as Cyclin E1 or Myc. We also note that the precise form of replication fork stress seen in FBXW7 mutant cells exposed to CDC7 inhibitor has not, as yet, been defined. DNA fibre analysis [53] could be informative in this regard, as could a detailed analysis of the effects on the composition, phosphorylation status and activity of the CMG complex in FBXW7 mutant cells exposed to CDC7 inhibitor.

## 5 Conclusion

Genome‐wide CRISPR‐Cas9 screens, focussed RNA‐interference screens, whole and phospho‐proteome mass spectrometry profiling and small molecule inhibitor experiments in multiple *FBXW7* wild‐type and defective isogenic cell lines identify a series of *FBXW7* synthetic lethal effects, including a RIF1 dependent *FBXW7/CDC7* interaction.

## Conflict of interest

CJL makes the following disclosures: receives and/or has received research funding from: AstraZeneca, Merck KGaA, Artios. Received consultancy, SAB membership or honoraria payments from: Syncona, Sun Pharma, Gerson Lehrman Group, Merck KGaA, Vertex, AstraZeneca, Tango, 3rd Rock, Ono Pharma, Artios, Abingworth, Tesselate, Dark Blue Therapeutics. Has stock in: Tango, Ovibio, Enedra Tx., Hysplex, Tesselate. CJL is also a named inventor on patents describing the use of DNA repair inhibitors and stands to gain from their development and use as part of the ICR “Rewards to Inventors” scheme and also reports benefits from this scheme associated with patents for PARP inhibitors paid into CJL's personal account and research accounts at the Institute of Cancer Research. ANJT reports personal honoraria from Pfizer, Vertex, Prime Oncology, Artios, MD Anderson, Medscape Education, EM Partners, GBCC conference, Cancer Panel, Research to Practise, honoraria to either the Institute of Cancer Research or King's College research accounts from SABCS, VJ oncology, GE Healthcare, Gilead, AZ ESMO symposium, IBCS conference, AstraZeneca Ad boards, honoraria and stock in InBioMotion, honoraria and financial support for research from AstraZeneca, Medivation, Myriad Genetics, Merck Serono. Travel expenses covered by AstraZeneca for any trial‐related meetings or trial commitments abroad. ANJT reports benefits from ICR's Inventors Scheme associated with patents for PARP inhibitors in BRCA1/2 associated cancers, paid into research accounts at the Institute of Cancer Research and to ANJT's personal account.

## Author contributions

JSB, RB, DBK, FS, SS, AG, JA, TIR, ZK, JSC, SH: data generation, data visualisation, data interpretation, writing and editing of manuscript. SJP, ANJT, CJL: project conceptualisation, supervision, data interpretation, writing and editing of manuscript. All authors approved the final version of the manuscript.

### Peer review

The peer review history for this article is available at https://www.webofscience.com/api/gateway/wos/peer‐review/10.1002/1878‐0261.13537.

## Supporting information


**Fig. S1.** Generation and characterisation of FBXW7^mutant^ cells.
**Fig. S2.** Chracterisation of the FBXW7^mutant^ cells.
**Fig. S3.** FBXW7 mutant cells are sensitive to CDC7 inhibition.
**Fig. S4.** FBXW7 vs. CDC7 synthetic lethality is RIF1 dependent.
**Fig. S5.** EdU foci analysis in DLD1 FBXW7−/− cells.

**Table S1.** Mass spectrometry whole proteome profiling in MCF10A p53^mutant^ clones with FBXW7 mutations described in Fig. 1.
**Table S2.** Mass spectrometry phospho‐proteome profiling in MCF10A p53^mutant^ clones with FBXW7 mutations described in Fig. 1.
**Table S3.** Gene Set Enrichment Analysis of the upregulated proteins in the total proteome analysis.
**Table S4.** Gene Set Enrichment Analysis of the upregulated proteins in the phospho‐proteome analysis.
**Table S5.** Gene Set Enrichment Analysis of the downregulated proteins in the total proteome analysis.
**Table S6.** Gene level *Z* score from the genome‐wide CRISPR‐Cas9 screen described in Fig. 2.
**Table S7.** List of the FBXW7 synthetic lethal genes.
**Table S8.** Gene Set Enrichment Analysis the FBXW7 synthetic lethal genes.
**Table S9.** *Z* scores from siRNA validation screens described in Fig. 3.

## Data Availability

CRISPR screen raw sequencing data is available on ArrayExpress under E‐MTAB‐12939 accession number and also described in the Supplementary Information. Mass spectrometry raw data is available on the ProteomeXchange repository with dataset identifier PXD043904. Mass spectrometry data is also described in the Supplementary Information.
