# Supplementary material for: Cancer‐associated FBXW7 loss is synthetic lethal with pharmacological targeting of CDC7
Source: Mol Oncol. 2023 Oct 22;18(2):369–85. doi: 10.1002/1878-0261.13537 (PMC10850818; doi:10.1002/1878-0261.13537)

Supplementary Figure 1.

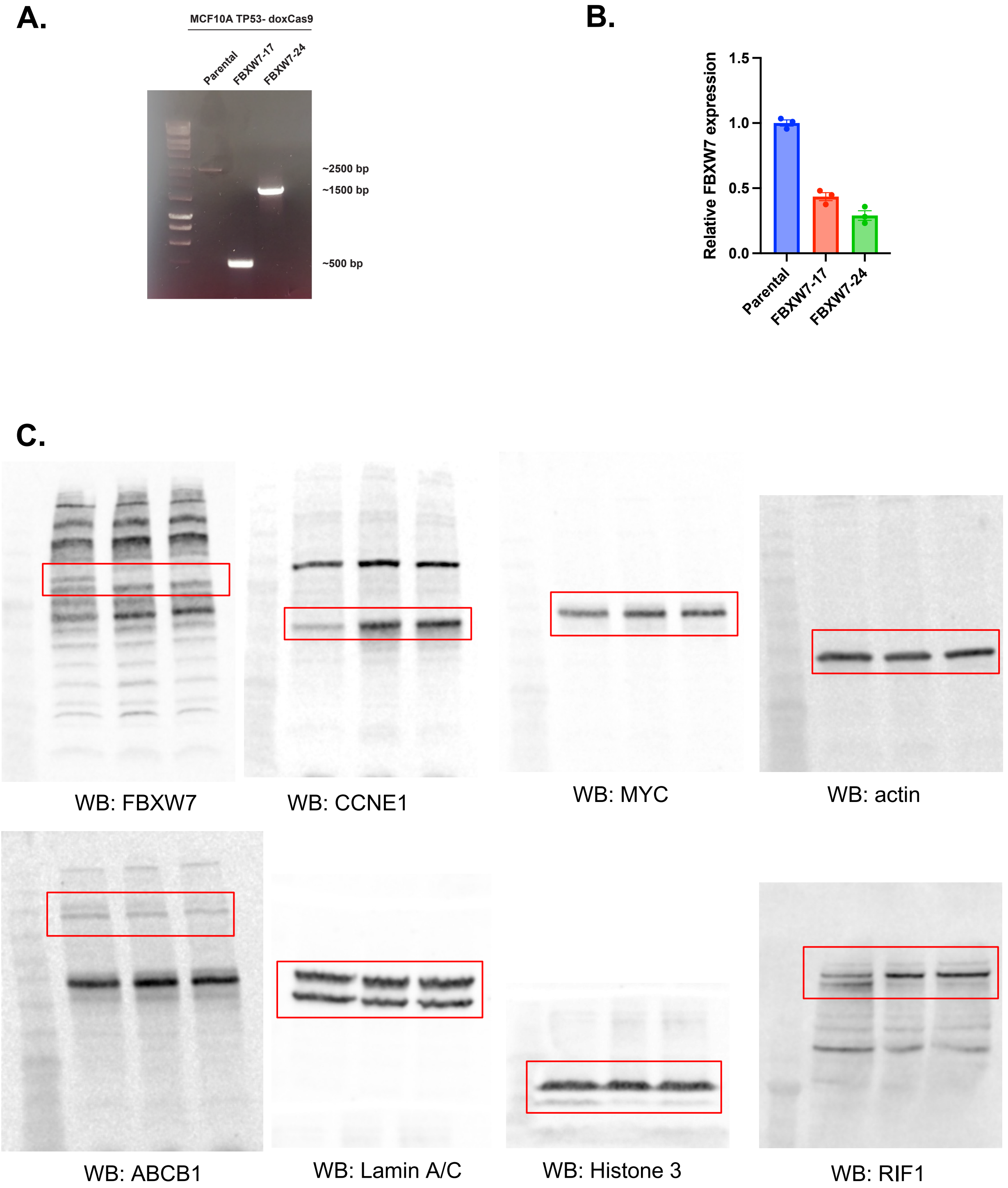

Supplementary Figure 1.

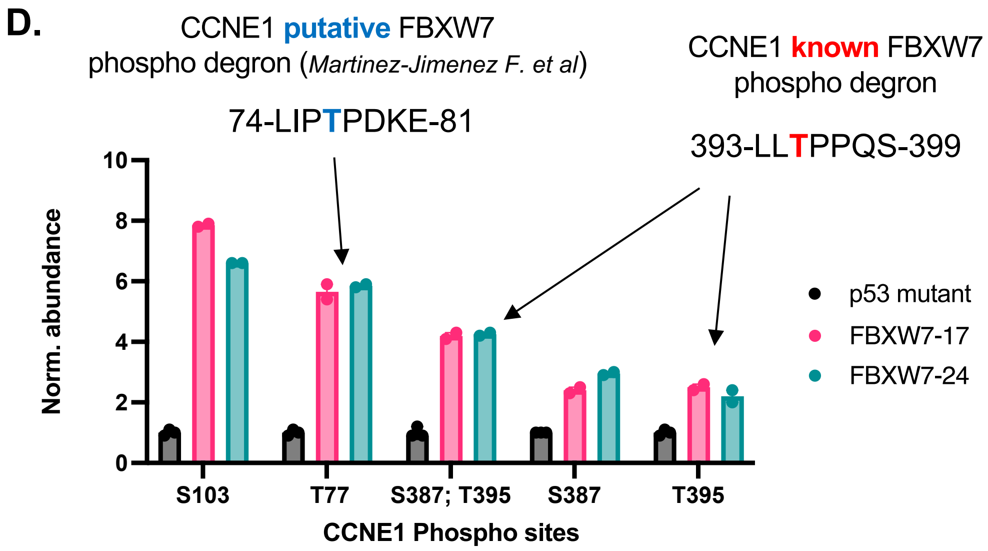

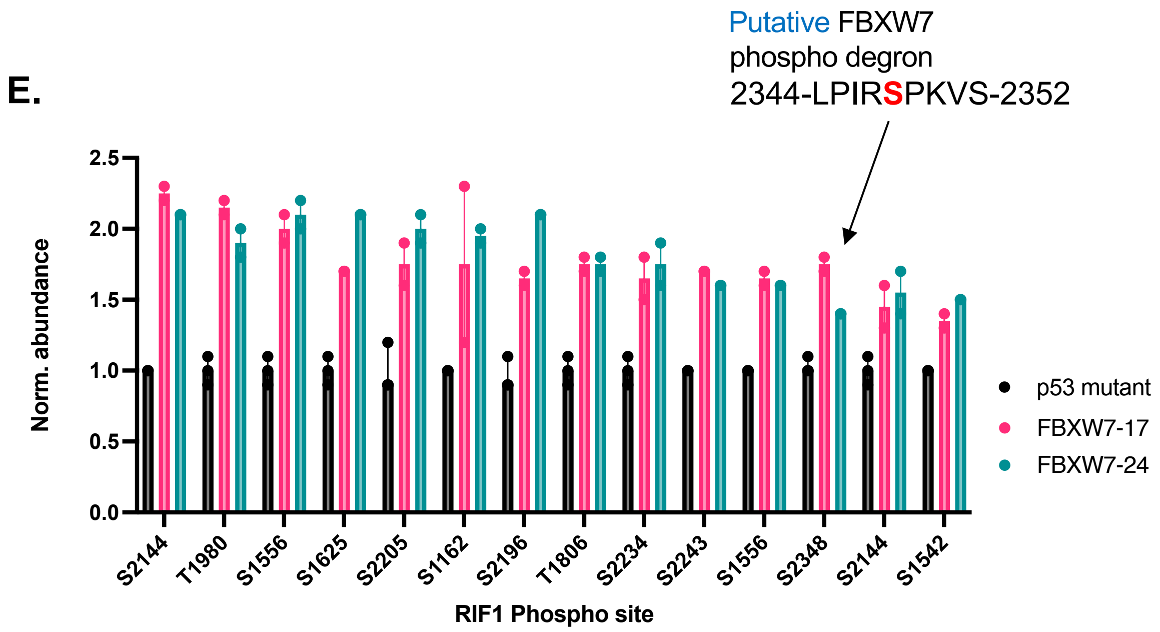

Supplementary Figure 2.

A.

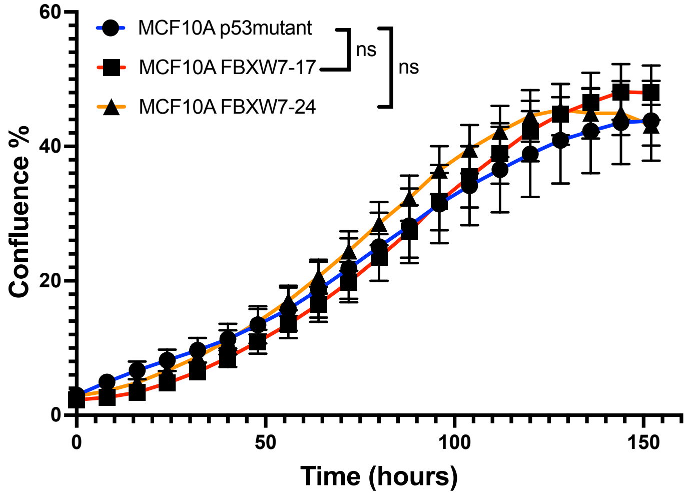

B.

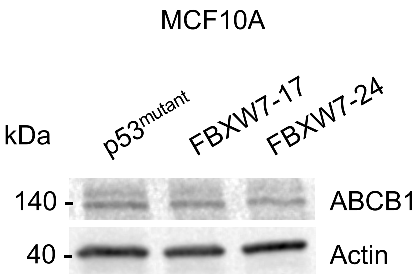

C.

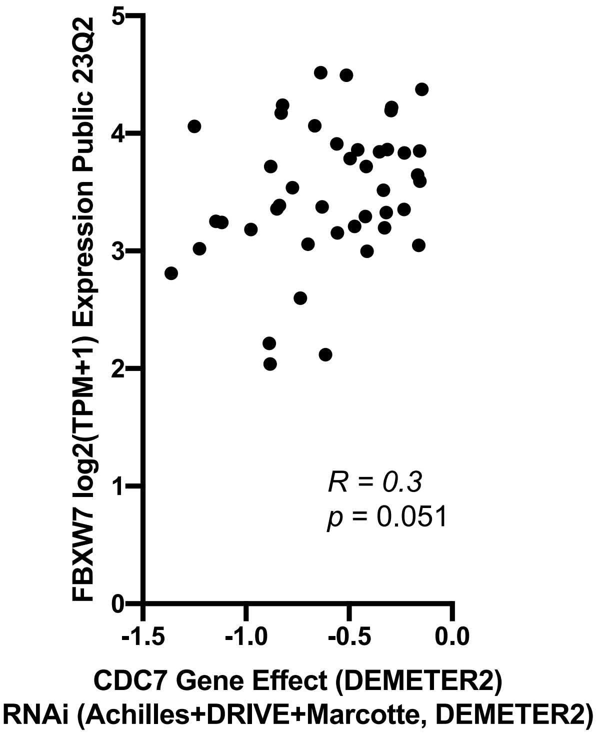

Supplementary Figure 3.

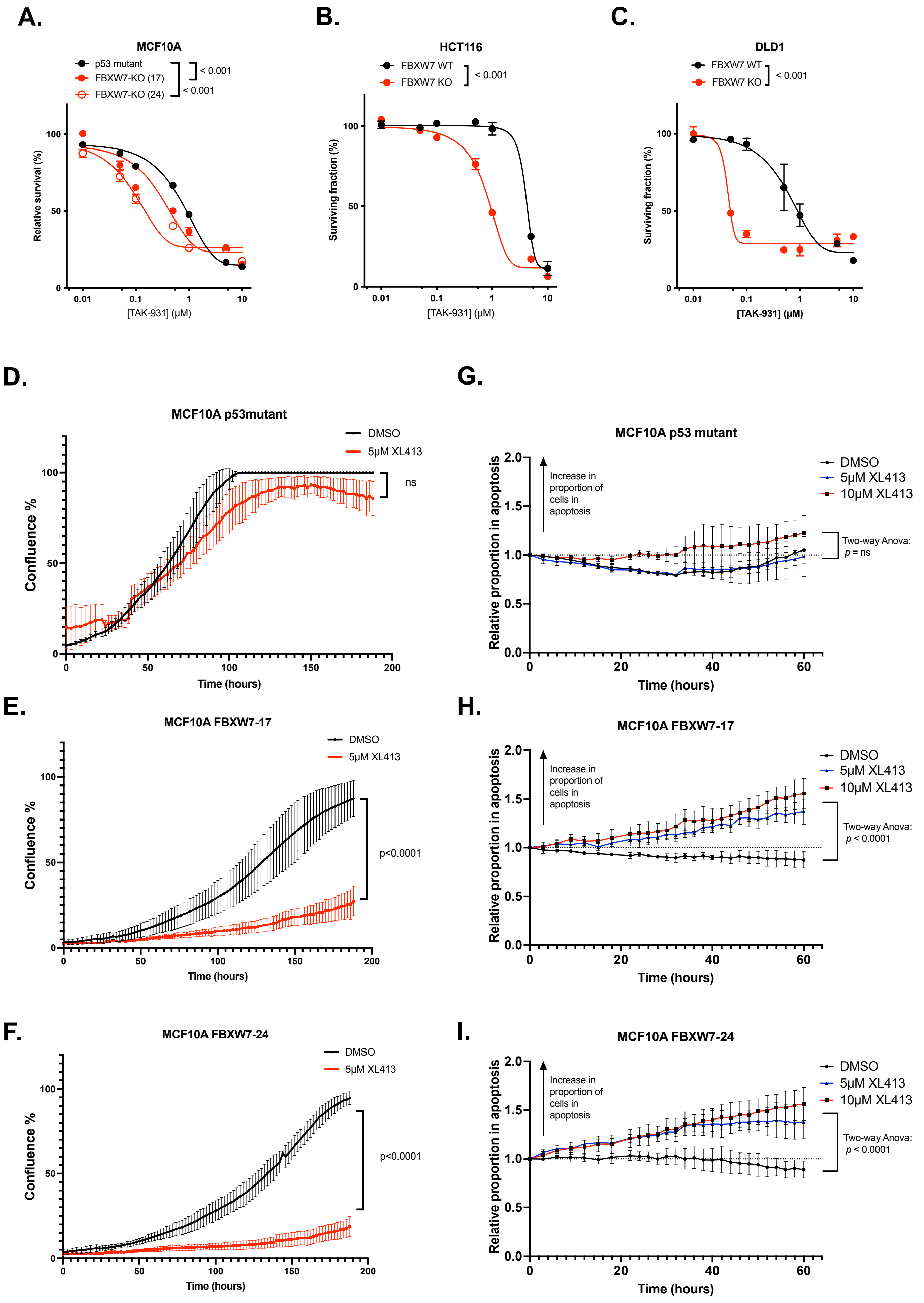

Supplementary Figure 4.

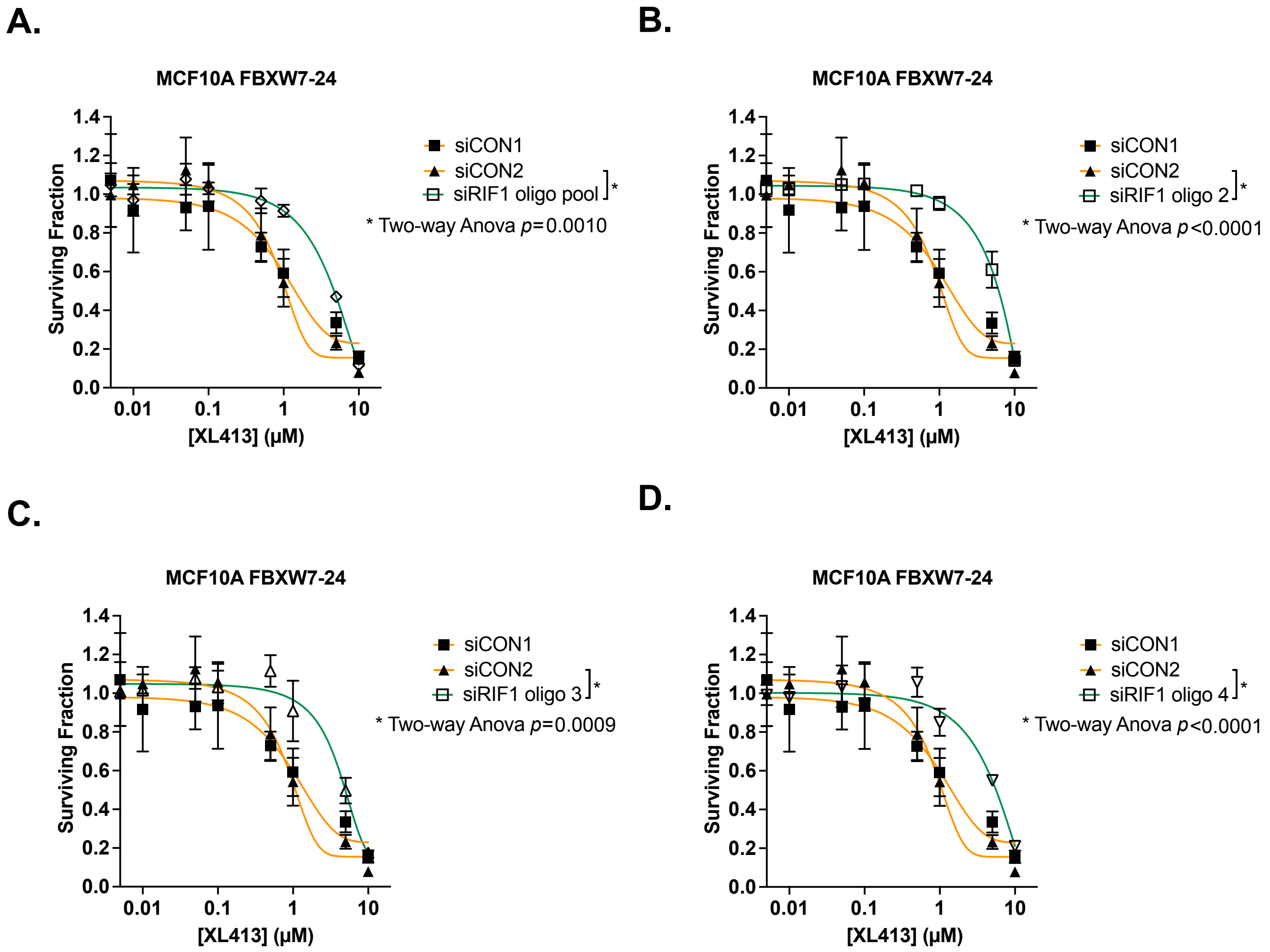

Supplementary Figure 5.

A.

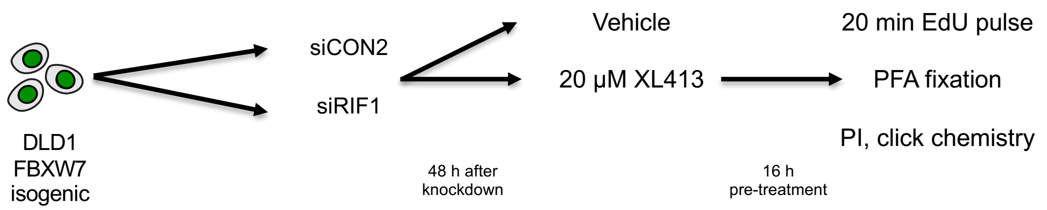

B.

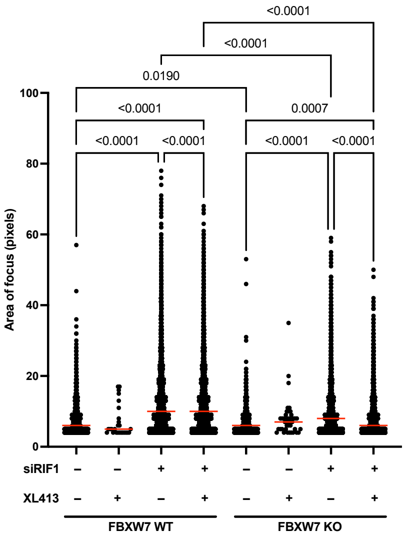

C.

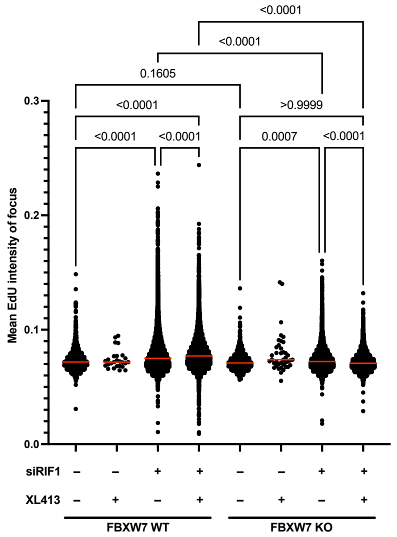

Supplement: Supplementary file 1 — Fig. S1. Generation and characterisation of FBXW7mutant cells. Fig. S2. Chracterisation of the FBXW7mutant cells. Fig. S3. FBXW7 mutant cells are sensitive to CDC7 inhibition. Fig. S4. FBXW7 vs. CDC7 synthetic lethality is RIF1 dependent. Fig. S5. EdU foci analysis in DLD1 FBXW7−/− cells. [file MOL2-18-369-s001.pdf]
